# Exploring the Lived Experience of Healthcare Discrimination and Resilience among Postpartum African American Women: A Qualitative Study

**DOI:** 10.1007/s40615-025-02620-5

**Published:** 2026-02-06

**Authors:** Melissa Neal, Katherine Quinn, Anna Palatnik, Erica Marion, Kiarri N. Kershaw, Joanne Lagatta, Jennifer Walsh, Kirsten Beyer

**Affiliations:** 1 Department of Family Medicine and Community Health, University of Wisconsin School of Medicine and Public Health, University of Wisconsin-Madison, Madison, WI USA; 2 Department of Psychiatry and Behavioral Medicine, Medical College of Wisconsin, Milwaukee, WI USA; 3 Department of Obstetrics and Gynecology, Division of Maternal-Fetal Medicine, Medical College of Wisconsin, Milwaukee, WI USA; 4 Department of Preventive Medicine, Northwestern University Feinberg School of Medicine, Chicago, IL USA; 5 Department of Pediatrics, Medical College of Wisconsin, Milwaukee, WI USA; 6 Division of Epidemiology and Social Science, Institute of Health and Humanity, Medical College of Wisconsin, Milwaukee, USA

**Keywords:** Healthcare discrimination, Resilience, Adverse pregnancy outcomes, Qualitative

## Abstract

African American women are at higher risk of adverse pregnancy outcomes (APOs) than women in other racial and ethnic groups in the United States, which can lead to an increased risk of maternal mortality and morbidity. These disparities have been attributed to inequities in social determinants of health. This study explores the experiences of healthcare discrimination and resilience among African American women. From December 2023 to August 2024, we conducted 30 semi-structured interviews with African American/Black (AA/BL) postpartum women in Milwaukee, Wisconsin, USA. The interviews included the following content domains: life experiences of pregnancy, adverse pregnancy outcomes, healthcare racism or discrimination during pregnancy, stress, and resilience. Interviews were transcribed verbatim and coded using MAXQDA 2024 qualitative software. Themes were generated using thematic analysis to understand how AA/BL women experience discrimination during pregnancy and childbirth and how resilience manifests in their lives. Two themes were identified: 1. Perceived healthcare discrimination during pregnancy and childbirth, and 2. Leveraging resilience to mitigate experiences of healthcare discrimination and life stressors. The results highlight that AA/BL women experience healthcare discrimination during pregnancy and childbirth, including poor quality care, and mitigate these experiences by seeking Black OBGYN providers. It is evident from the stories in our study that AA/BL women are highly resilient and display positive coping mechanisms to overcome challenges in their lives. While resilience can help to buffer against adversity, systems and policies should be in place to protect against stressors.

## Introduction

African American/Black (AA/BL) women face the highest maternal mortality and morbidity rate in the United States [1, 2]. In 2021, AA/BL women had 2.6 times the maternal mortality rate of White women [3]. Key drivers of these disparities are adverse pregnancy outcomes (APOs), which include a variety of complications (e.g. preterm birth, small for gestational age, hypertensive disorders of pregnancy, gestational diabetes) that can occur during pregnancy and childbirth, potentially leading to unfavorable cardiovascular outcomes later in life [2, 4, 5]. AA/BL women have been found to have higher rates of APOs compared to other socially constructed racial and ethnic groups [6–8]. Moreover, hypertensive disorders of pregnancy (HDP) and preterm birth (PTB) are the two most prevalent APOs among AA/BL women [6–8]. The risk factors underlying racial disparities in HDP and PTB are complex and multifaceted. Significant evidence suggests that inequities in social determinants of health, such as discrimination, are leading contributors to health disparities [9–11].

Experiences of discrimination have been defined as a chronic social stressor occurring across an individual's life, leading to adverse physiological effects and future negative health outcomes [12–19]. Discrimination perpetuates societal power imbalances and can be characterized as negative interactions or attitudes toward an individual of a particular group based on individual characteristics, such as race [12, 14, 17, 19]. Experiences of discrimination can occur at the structural, institutional, internalized and/or interpersonal levels [12, 14, 20]. Discrimination is not always overt and can often manifest in subtle ways, such as showing a lack of respect, stereotypes, believing that a person or a group are suspicious because of their race, and not listening to concerns [21]. Furthermore, subtle discrimination, often defined as microaggressions, have been suggested to cause more harm than those that are more overt [12, 14, 19, 21–24]. In pregnancy, experiences of discrimination can lead to psychosocial impacts, which have been found to trigger physiological changes in the body, that are hypothesized to lead to APOs among AA/BL women [25–28].

Healthcare discrimination has been identified as a major source of discrimination among AA/BL women [29, 30]. Studies have consistently shown in qualitative research that AA/BL women experience discrimination through poor communication, provider disrespect, judgment, and bias [31–33]. Additionally, prior research has underscored healthcare provider bias in the way they provide care to AA/BL women. A prior study highlighted implicit bias among providers who ordered more drug screens for Black women compared to White women [34]. This study also found that when it came to pain management, providers were more concerned with symptom exaggeration for Black women than with overdose for White women [34]. These findings were consistent with prior studies that found differential treatment based on race and socioeconomic status [35, 36]. Differential treatment has been shown to negatively impact how AA/BL women engage in healthcare [36, 37]. One recent study found that Black birthing people had a preference of racial concordance with their obstetricians and midwives as a result of racial discrimination throughout their lives and a fear of death [38]. Despite a growing body of research indicating that AA/BL women experience discrimination in healthcare during and after pregnancy, further qualitative research could provide more insight into these experiences among AA/BL women and how they cope with them.

Resilience, an individual's ability to process and overcome adversity, and its influence on APOs is a growing area of research. Previous research has highlighted the protective role of resilience on reducing stress in pregnancy [39, 40]. However, studies on resilience during pregnancy and its influence on APOs are limited [41, 42]. Grobman et al. (2018) found no significant association between resilience and PTB or small for gestational age [43]. However, several studies have suggested that lower resilience is associated with a higher likelihood of APOs [42, 44]. One study outside of the U.S. found that women with high-risk pregnancies (e.g., diabetes with pregnancy, hypertensive with pregnancy, bleeding in early pregnancy, placenta previa) who had higher resilience were less likely to have complications in pregnancy than those with lower resilience [45]. However, research has suggested that resilience may not be as protective in eliminating disparities in pregnancy outcomes among AA/BL women [46]. The risk-adjusted rate of low birthweight was higher among highly resilient AA/BL women compared to highly resilient Hispanic and White women. These findings were also confirmed by other studies that found that resilience did not change racial disparities in PTB and other APOs [43, 47]. These findings may suggest that resilience may not be enough to combat racial disparities in APOs. Additionally, it may also indicate that other factors such as experiences of discrimination may play a role in the relationship between resilience and APOs. Research has suggested that in the lives of AA/BL resilience may be shaped by their experience of historical oppression and structural vulnerability [48, 49]. Additionally, it is hypothesized that AA/BL women demonstrate resilience through the “strong black woman” or “superwoman phenomenon” [49, 50]. The Superwoman Schema (SWS) framework was created to examine the “strong Black woman” or “superwoman” phenomenon, focusing on how AA/BL women cope with and withstand race and gender based oppression within sociopolitical and historical contexts [49–51]. Therefore, it is possible that resilience may operate differently in the lives of AA/BL compared to other racial and ethnic groups and traditional measures of resilience may not fully capture the culturally relevant resilience factors such as spirituality, strength and social support [52]. This study seeks to explore how resilience manifests in the lives of AA/BL women.

Considering the prior research that has highlighted the experiences of healthcare discrimination among AA/BL women, understanding these experiences and how AA/BL women bounce back from these challenging situations is imperative. To our knowledge, only one qualitative study has sought to explore experiences of healthcare discrimination and how AA/BL cope with these experiences using an intersectionality framework [53]. However, no studies have explored these experiences by pregnancy outcomes. Our study is unique in that we explore lived experiences of healthcare discrimination and resilience among AA/BL women with different pregnancy outcomes. Stratifying our sample by pregnancy outcomes is critical to identifying if there are differences in healthcare discrimination experiences and resilience across pregnancy outcomes. Differentiating experiences by pregnancy outcome can help to inform tailored strategies for prevention and quality care improvement. Thus, additional qualitative research can help to provide a more comprehensive understanding of how healthcare discrimination may shape maternal health and its psychosocial impacts among AA/BL women with different pregnancy outcome experiences.

## Methods

### Reflexivity and Positionality

This study took a reflexive approach to analyzing the semi-structured interviews. We recognize that researchers'identities influence the research questions posed, the analysis of data, and the interpretation of qualitative findings. The current study was led by an AA/BL cisgender woman with lived experience of preterm birth. The rest of the team contributes expertise in qualitative data analysis, research on racial disparities, and clinical knowledge in obstetrics and gynecology, and maternal–fetal medicine. The diversity of our research team fostered in-depth discussions about the data, the implications of the findings, and the context for understanding women’s'experiences.

### Study Design

This study aimed to explore the lived experiences of healthcare discrimination and resilience among AA/BL postpartum women in Milwaukee, WI, USA. Eligibility criteria included 1) Self-identified as African American or Black; 2) 18 years or older; 3) delivered in past 24 months; 4) singleton live birth; 5) the ability to provide informed consent.

### Interview Guide

A semi-structured guide was used to facilitate the interview. The research team explored the literature and received feedback from the senior qualitative researcher before finalizing the interview questions. The content included the following domains: experiences of pregnancy, life leading up to pregnancy, experiences of prenatal care, experiences of preterm birth, experiences of hypertensive disorders of pregnancy, experiences of racism or discrimination in healthcare during pregnancy, and experiences of stress and resilience.

### Data Collection

To enhance diversity in the sample, AA/BL women were recruited using a purposive sampling strategy, with recruitment stratified based on pregnancy outcomes. Between December 2023 and August 2024, we conducted 30 semi-structured interviews with AA/BL postpartum women. Women were recruited in-person in a level four birth center at an academic medical center. AA/BL women who delivered were screened via electronic medical records for eligibility by the study team. Thirty eligible AA/BL women (10 women with HDP, 10 women with PTB, and 10 women with no APOs) were invited to participate in the semi-structured interview via Zoom or phone with a trained qualitative researcher. Prior to an interview, individuals received an informational letter explaining the goals of the study, risks, and benefits. Women provided verbal consent to participate and received a $25 Visa gift card for participation in the semi-structured interview. The study was approved by the institutional review board at the primary author’s academic institution.

### Analysis

Interviews were audio recorded and transcribed verbatim for data analysis. The initial codes and codebook were developed using deductive coding from the interview guide and an inductive approach from transcripts (e.g. fertility, unplanned pregnancy, fears about high-risk pregnancy). The initial codebook was applied to ten transcripts and revised between the primary author and the research team’s senior qualitative researcher. Using the final codebook, all interviews were coded using MAXQDA software. Following coding, the team used thematic analysis to identify experiences of healthcare discrimination that women reported and sought to understand how women coped with those situations [54]. Furthermore, we analyzed the data to identify how resilience manifested in the lives of AA/BL women. Our analysis included an iterative process to develop and finalize themes to answer the following research questions:

1. What are AA/BI women’s experiences of healthcare discrimination during pregnancy and childbirth?
2. How does resilience manifest in the lives of AA/BL women during pregnancy and childbirth?

## Results

### Participant Demographics

Table 1 presents the demographic characteristics of 30 AA/BL women who participated in semi-structured interviews. The average age of the women was 30 years (range: 19–43). The majority of women (73%, *n* = 22) reported their relationship status as single. Thirty percent (*n* = 9) of the women had a high school diploma or equivalent, 43% (*n* = 13) had some college credit, and 13% (*n* = 5) held a college degree. Seventy percent (*n* = 21) were employed, 13% (*n* = 4) were unemployed, and 7% (*n* = 2) were unable to work due to illness or disability. Thirty-seven percent (*n* = 11) earned less than $20,000 a year, 17% (*n* = 5) earned between $20,001 and $40,000 a year, 20% (*n* = 6) earned $40,001 to $60,000 annually, and 13% (*n* = 4) earned $60,000 or more per year. Seventy-seven percent had Medicaid insurance, and 79% (*n* = 23) received prenatal care in the first three months of pregnancy. Thirty-three percent (*n* = 10) had no APOs, 30% (*n* = 9) had PTB, 33% (*n* = 10) had HDP, and one woman had both HDP and PTB.

**Table 1:** Demographic characteristics *N* = 30

Demographic variable	N (%)
**Age mean(sd)**	30.43 (6.68)
**Marital status**	
Single	22(73.3%)
Married or living as married	7 (23.3%)
**Income**	
Less than $20,000	11(36.7%)
$20,000 to $40,000	5 (16.7%)
$40,0001 to $60,000	6 (20.0%)
$60,0001 or more	4 (13.3%)
**Employment**	
Employed	21 (70.0%)
Unemployed	4 (13.3%)
Unable to work due to disability	2(6.7%)
**Education**	
No high school diploma	2 (6.7%)
High school graduate	9 (30%)
Some college	16 (53.3%)
Bachelors	2 (6.7%)
**Insurance**	
Private insurance	6 (20%)
Medicaid	23 (76.7%)
**Prenatal care in first trimester**	
Yes, prenatal care in 1–3 months	23 (79.3%)
No, prenatal care in 1–3 months	4 (13.8%)
No prenatal care at all	2 (6.9%)
**Pregnancy outcomes**	
PTB	10 (33%)
HDP	10 (33%)
HDP and PTB	1 (3.3%)
No APO	9 (30%)

Table 1 presents the demographic characteristics of the 30 African American/Black women who participated in qualitative interviews. *PTB* Preterm Birth, *HDP* Hypertensive Disorders of Pregnancy, *APO* Adverse Pregnancy Outcome

### Thematic Results

Our data are presented in two primary themes that illustrate healthcare discrimination through inadequate quality of care and patterns of resilience.

#### Theme 1: Manifestation of Healthcare Discrimination During Pregnancy and Childbirth

As part of the interview, all women were directly asked about experiences of discrimination in healthcare settings during pregnancy and childbirth. Many AA/BL women in this study, regardless of their pregnancy outcome, discussed experiences of discrimination or mistreatment while receiving care during pregnancy and childbirth. Most commonly, these experiences were not explicitly racist but were experienced through poor quality of care and mistreatment that were interpreted by the women as discriminatory. Women reported negative interactions with a variety of staff members, including physicians, nurses, phlebotomists, and others they encountered during healthcare visits during pregnancy. While some of these experiences [e.g., failure to do certain procedures or processes] may have had clinical justifications, women often experienced them as discrimination and racism enacted via inadequate medical care or dismissal of their medical needs. Perceived discrimination manifested through poor quality of care, including unfair treatment, dismissal of health concerns, poor communication, and lack of empathy from providers.

The first way women in this study experienced discrimination was through unfair treatment, particularly by nurses, during and after childbirth. When asked “can you tell me about a time you were mistreated or discriminated against and didn’t get the care you deserved?”, one women vividly reflected on the differences in care between her daytime and night nurse. The women described her night nurse as “cold” and “not hands on.” The nurse failed to support her after delivery, leaving her to struggle to the bathroom while she was on medicine to induce her labor.

I had a really, really great day time nurse. She was amazing. The care during the labor and delivery for the night, I had a nurse, she was so not hands on, just cold...even during the whole process, making me do everything for myself; she didn’t attend to my needs, whatsoever. I felt, you know, the most vulnerable time in your life for a female. And I felt she just didn’t care. I just felt like another number. You know, I had to get up to go to the bathroom, she up my dose of pitocin. And I had to go to the bathroom and just... I was looking for assistance to get out of the bed and she was just like, well, I’ll be right here when you get out the bathroom, like, I’m struggling to walk my feet hurt, or my feet are swollen. And she just looked at me. I’m just like, okay, I literally said that. And I just got up and went to the bathroom, holding my gown, with the IV mind you. It was like 3am in the morning or whatever. So I'm struggling. And then I come out the bathroom. And my husband, he was sleep at the time. But then he had woke up he saw me coming out the bathroom. He assisted me to get back into bed. She wasn’t even in the room anymore. -(Interviewee 18, No APO)

Secondly, overwhelmingly, when asked about healthcare discrimination or mistreatment due to race or skin tone during pregnancy, women described experiences of dismissal of health concerns and not being listened to by healthcare providers. These experiences often led to fear and stress about the outcomes of their pregnancies. One woman who delivered preterm expressed not being listened to and experiencing mistreatment by a nurse during delivery. She explained that her daytime nurse was “kinda rude” and did not listen to her concerns of needing to push. She later went on to explain that it was a second shift nurse who confirmed that it was time to deliver. The women attributed the first nurse’s dismissal of her needs to the nurse’s shift ending and thought that the nurse put going home over providing adequate care. Some women attributed the substandard care they received to being Black. One women with HDP recalled their negative experience with a phlebotomist during birth. While clinically, IV placement for HDP patients can be hard, the patient explained that the phlebotomist did not listen to her complaints of pain and ultimately, did not “do it right,” which resulted in another phlebotomist coming in to do the IV. The women felt their interactions were “kind of weird.” She went on to say, “I feel like they wouldn’t have done it to a Caucasian patient.”

Another women shared a similar experience she had with her husband when seeking care at the emergency department for a possible miscarriage. The women explained that she requested her husband stay in the room because the ultrasound technician was male, which made her uncomfortable. The nurse refused to let her husband stay in the room, and ultimately elevated the situation to the sheriff and hospital security when the patient and her husband spoke up. The women went on to say how they will “never forget” how they thought they were losing their baby and had to experience that mistreatment when seeking care. The women stated later in the interview, “I definitely do feel like race was a factor into it.” She shared her story:

I was like, well, ma'am, you know, I'm not comfortable with this man [ultrasound technician] down here, and I'm a woman and being in there by myself. And she's like, well, he [husband] needs to go back. That's our policy. And I was just like, Well, I'm not comfortable. Can you all have someone else come back? She's like, we don't have anyone else that can go back there with you. And she basically was like, if he doesn't go sit down, you all can go. She was like, “Y'all can leave.” And I was just in shock. So I'm sitting there in shock and my husband sitting there in shock. And we're both like damn. And then she proceeds to the sheriff that was there and say, like, we were causing an issue... the lady took the situation way out of proportion. She used intimidation with the security with the hospital, at first it was the sheriff's. The sheriff's saw that it was wrong. And they backed off. It was like this is a really unnecessary situation for us to be involved in. And then she when she saw that they weren't going to do anything. She utilized the hospital security to try and come intimidate us again. And so I did end up filing a complaint with her, the nursing department, the security department, but it was just a really awful experience at that campus...But it wasn't so it was just a really horrible it was just horrible. I will never forget that. I will never forget that lady saying “well, you can leave,” and I'm sitting here thinking I'm losing my baby. You know? Yeah, it was just awful... -(Interviewee 1, No APO)

In this narrative, the nurse unnecessarily escalated the situation to a sheriff and hospital security despite no real threat. The escalation to involve the sheriff underscores the systemic racism that the Black community often encounters with institutions and law enforcement. Furthermore, this women’s narrative displays how practices such as using the sheriff or security can disproportionately impact the care and mental well-being of Black women and their families.

When asked about experiences of discrimination, women expressed how dismissal of concerns resulted in adverse outcomes after childbirth. One women, quoted below, with no APOs, described her experience of having her concerns about heavy bleeding “shrugged off” by a nurse. She explained:

Well, there was a time like with my last pregnancy. I was experiencing large clots after birth... I feel like she [nurse] just shrugged it off, like, ‘Oh, that's normal.’ No concerns... And she was like,"Oh, that's normal” kind of thing. Like, I literally had blood drip from the bed to the bathroom... So when the next nurse had came in, I let her know, and then that's when she had grabbed, like a towel from before, like when I actually started bleeding and stuff like that, how much blood I lost, or whatever... she basically said I was hemorrhaging... So they had gave me some meds, like IV meds... They put a catheter in so that I didn't have to go to the bathroom or I wasn't moving that much.- (Interviewee 26, No APO)

In reflecting on the experience, the women stated, “I feel like if I was a different color, maybe it would have been a bigger concern, you know?” Another women described a time when her concerns were similarly ignored, resulting in complications during delivery. She went into the hospital for pain and a feeling that “something was wrong.” Despite her concerns, she was sent home, noting of her provider: “She kept saying, I can’t just give me anything for pain,” explaining that she thought “she just did it because I was Black or because she felt I wanted to get high.” As a result of being sent home, she lost a significant amount of blood and experienced complications, including the removal of her uterus. However, she explained due to another doctor listening to her concerns when she went back to the hospital and through life-saving efforts, her and her baby survived.

Like if you would have listened to me six hours ago, we wouldn't even be in this predicament, like we would have been delivered the baby. We would have found out what was wrong, if you would have even just sent me back for an ultrasound, like I asked, because, again, this wasn't my first baby. I'm telling you. Something is wrong. Take me back and figure out what is wrong, like and I just feel like she should have heard me as a patient, and she didn’t. She said, screw how I felt, I didn't know my body. Like you're trained as a doctor. You're learning as a doctor. That is, to me, that is general medicine. I'm speaking as a patient. This is my body. Nobody knows my body like I know my body. So that was just terrible for me. I will never forget it.... she changed my life forever. I don’t have a uterus. Like, okay, I won't have a period anymore at 32 years old. That'll forever live in my head. It is something that I'll never forget, and I probably will never forgive for that. -(Interviewee 28, PTB)

In this excerpt, you can hear the grief that the women has over losing her uterus. The description of her hurt reflects what many AA/BL women in this study described. Furthermore, it highlights the importance of listening to AA/BL women and the impact it can have on pregnancy and birth outcomes.

Finally, the third way discrimination during pregnancy and childbirth manifested in the lives of AA/BL women was through poor patient-provider communication. Namely, this was evident in poor explanation of important information and lack of empathy among providers. Several women expressed frustration around receiving a lack of information and lack of sensitivity regarding diagnoses during pregnancy. They described experiences of healthcare providers failing to properly explain diagnoses, procedures, and potential pregnancy complications. One women, when asked about experiences of healthcare discrimination, explained that “no one was communicating what was going on” when they were in labor. This lack of communication led to a feeling of hopelessness and fear about death during delivery. As they were prepping for delivery she asked, “Am I gonna die?”.

Another women associated perceived discrimination with a provider's lack of empathy when communicating about their high-risk ultrasound, and felt it was “somewhat because of my race.” She recounted feeling shocked when a healthcare provider lacked empathy regarding potential of fetal death. She stated the doctor “immediately started off…talking about death” when explaining the results of their ultrasound rather than explaining the diagnosis and the solutions. It was evident in these women’s stories that experiences of poor-quality care were often perceived as discrimination.

#### Theme 2: Leveraging Resilience to Mitigate Experiences of Healthcare Discrimination and Life Stressors

As a result of healthcare discrimination AA/BL women leveraged resilience to mitigate these experiences and day to day life stressors. When asked how women “bounced back” from challenging experiences or what they did about the perceived discrimination during pregnancy or childbirth, AA/BL women described ways they took action to address their experiences. Commonly, AA/BL women filed formal complaints and spoke to their healthcare providers about their experiences. One woman below described how making a complaint and her provider’s action helped her “recover” and have a positive outlook that “everybody isn’t bad.”

Additionally, she described how speaking up and sharing her story may help to prevent this from happening to other mothers.

And that helped me to just say, “everybody isn't bad, right?” Like everybody isn’t bad, this is gonna be okay. So that immediately helped me start to recover... I also I think being able to share this story and talk to you, as well, because it's like, what, how can we prevent this from happening to other people? And that was the most important thing to me. Like, how can I prevent this from happening to another mom?- (Participant 1, No APO)

Another way that we conceptualized women coped with healthcare discrimination was by seeking Black healthcare providers during pregnancy, stating the need for cultural understanding. Women spoke about patient-provider racial concordance saying, “I felt more comfortable with someone of my, you know, my culture” and expressing desire for a doctor who “understands me more, understand me better.” Another women further explained:

Not to sound racist or anything, but I prefer African American doctors, because I feel like they understand me more, they understand me better. And what my doctor been African American, and then even my primary doctor is African American, I feel like they have a sense of who I am. - (Interviewee19, HDP)

Women said that seeking a Black OBGYN helped to mitigate healthcare mistrust and was associated with better quality of care during pregnancy. One woman stated that her prenatal care was a 10 because she was “adamant about finding a Black doctor.” The women highlighted the notion of a shared understanding with the provider of the inequities faced by Black women and how important it was to “find someone who looked like me and could relate to these issues.”

In addition to a shared understanding, AA/BL women felt that Black OBGYNs and providers were more likely to listen to their concerns during pregnancy. One women below discussed seeking a Black OBGYN because she heard that other doctors don’t listen to Black women.

I thought it would be nice to have a Black woman as an OBGYN, just because I was scared, as far as delivery. I was hearing like that other doctors don't listen to Black women and their concerns in the delivery room. So yeah, I did choose her because of race. - (Interviewee 24, PTB)

Another women, quoted below, had the same experience. She felt that her Black OBGYN was more attentive to health concerns and went the extra mile, listened, and tried to figure out what was going on.

I had my primary care physician she was White. Like, the experiences that I had with her, it wasn't like the old school way... I would tell them about my thyroid. And they, be like “Oh, well that’s something else.” But me knowing what it is and my body. I'm like, well, it’s this. So then I went to my OB, and I tell her like, I'm experiencing hip pain. And then she was like, “Well, maybe we need to do ultrasound and see what's going on.” We do the ultrasound, she’s like oh that's why, the baby is laying in your hip. So it's kind of like that. Listen to me if I'm telling you something, I want you to like least try to figure it out...-(Interviewee 15, No APO)

Other women similarly shared how they felt that their concerns during pregnancy were taken more seriously by Black OBGYNs. One woman expressed concerns over potentially losing a baby and felt “she [Black OBGYN] didn’t dismiss any question.” She discussed below how her Black OBGYN took her concerns into consideration and did not dismiss questions in comparison to another provider of a different race, noting that she “could tell the difference” between the two experiences.

This was my first baby. And then I had those concerns about losing my baby. And then you know, with me being overweight and stuff and having those concerns about being high risk. Her knowledge and her experience definitely was helpful for me. And she also took my concerns into consideration. Like, she didn't dismiss any question I had, even with my husband, you know, she didn't dismiss his questions, either. I'll give you an example, she did let me know, “Hey, you may have to work with the other providers that I work with, but if there's something you're not comfortable with, you need to let me know.” I did see one of her colleagues. And during my visit with him, I could tell the difference. - (Interviewee 1, No APO)

AA/BL women in our study experienced discrimination because of poor quality care and mistreatment, and to prevent these experiences, some women sought Black OBGYNs. In this final section, we detail how AA/BL women described overcoming challenges (e.g., stress and discrimination) in their lives overall. We asked women about how they got through difficult times in their lives to understand how resilience and strength might be harnessed to support AA/BL women.

The strength and will to “fight” against challenges were commonly expressed across interviews. AA/BL women commonly discussed facing challenges head on, getting through challenging times by never giving up, and continuing to keep going regardless of life stressors. One woman displayed strength and tenacity as she “fought” through challenges:

Don't give up, I don't lay down, I'm a fighter. My challenges my biggest challenges and I will say as strong as I am I am a cry baby but once I'm done with the emotional part of it, I get back up and fight. -(Interviewee 11, HDP)

Resilience was bolstered through optimism and keeping a positive outlook. When asked “I would love to hear a little bit more about how you got through those hard times and what got you through it?” two women stated:

I feel like just knowing that everything will be okay, kinda is what got me through like, I just really know and feel like everything will work out and be okay... - (Interview 8, HDP)

I just keep going with life. Life be life’n it. I have to realize that. I try to stay positive. But sometimes it's just like, alright, nothing to go right for me...everybody has their moments. So I have to realize, some people have worse problems than what I'm going through....some people forget that, like it's people out here that's way worse. -(Interviewee 14, PTB)

Furthermore, there was a sense of not having a choice but to keep going. One women discussed not being able to stop because she is her children’s “only source,” so she has to “figure out a way to keep it going.” Another women below discussed not stressing about it and trying to “keep myself going.”

If I can't change it, I don't stress about it. I try to use that to keep myself going. I feel like every day that I wake up, that's God's way of giving me another chance to get it right. So I typically try not to stress, especially because stress is a silent killer. So I try not to stress. I have too many people counting on me to take myself out due to stress. -(Interviewee 28, PTB)

It was evident in this passage and many others that women had to keep going because it was a way of life and have to deal with it. The concepts of strength and determination have been explored in the literature as the “strong Black woman” or “superwoman” phenomenon and have been hypothesized as one way AA/BL women demonstrate resilience. Additionally, as a result of historic oppression, it is believed that AA/BL women are highly resilient [55]. It was evident in this study that AA/BL women developed resilience throughout their lives as a result of challenges in life. Patterns of superwoman phenomena came through when discussing resilience and overcoming challenges. It was evident through interviews that AA/BL women prioritized being strong and independent in challenging situations. One women described herself as a “perfectionist” and when asked if she feels like if the burden is on her, she explained even with help, she naturally likes to get things done and is “very much so independent.”

Additionally, AA/BL women prioritized the needs of their children. When asked “What would you say are your greatest strengths or characteristic things about yourself that help you overcome challenges or in hard times?” One woman stated, “you have to be strong… when you have kids”.

Others similarly explained that motherhood was a significant source of strength and determination for AA/BL women. Children helped push women and motivated them to “keep striving for the better.”

Yeah, my kids are my motivation. So, if I feel like I want to give up, I think about them. And what they need are and what they, you know, what they want to do and everything. So, it's like, I gotta keep striving for the better. I can't give up- (Interviewee 9, HDP)

I guess my biggest motivation is... me taking care of my own kids raising them so they won't go through the same thing I went through as well as breaking the cycle. I always been big on breaking the cycle, I tell my kids as well, anything mommy go through you make sure you do better in life so you won't go through with it as well, that’s my motivation- (Interviewee 11, HDP 106)

In these excerpts one can hear women’s strength and determination to “break the cycle” for their kids and overcoming challenges in their lives. These descriptions of how motherhood is a major driver “to be better” and to keep going reflects the resilience described by many of the women in the study. However, it was evident in the voices of women that this resilience, “doing for their kids,” and being a superwoman took sacrifices. When women explained how they overcame challenges in their lives, they expressed the emotional toll that it takes to be “a fighter” and to “keep going in life.”

AA/BL women in the study shared common coping mechanisms to help to overcome challenges and stressors in their lives. These coping mechanisms included walking, journaling, listening to music, taking time for themselves, and talking to others. Additionally, there were some women who discussed the importance of organizing and finding solutions to the problem. In addition, spirituality also played a major role in AA/BL women overcoming challenges. Women expressed that praying and trusting in God also helped them get through hard times and feel better. One woman stated that “going to church just makes you feel better about everything.” Another woman stated:

I don't worry... I believe in God, so really I didn't worry, I was always praying...So I just feel like God really bless people, like, he really helped people. Like, when I look at my life now versus then how I grew up, like, I just really feel like I came a long way that’s God, God helped me get here. -(Interviewee 22, PTB)

In addition to positive coping mechanisms, social support was a major resilience resource among AA/BL women in our study. Social support in AA/BL women’s lives was commonly defined as having help with their children, having someone to talk to, emotional support, and being there when they needed help. Additionally, AA/BL women commonly discussed the important roles played by their mothers and grandmothers during pregnancy and the postpartum period. Overall, social support from family and friends played a significant role in AA/BL women overcoming challenges and stressors in their lives. One women said about social supports in her life:

Definitely helped. They always listened to my concerns. One of my cousins is like, she was a nurse at the time, so yeah, so that helped a lot. Like she listened to me, she explained, like, what I was feeling, or what it may be, or if I should go in. So yeah, it definitely helped a lot. Like, with stress, being pregnant. -(Interviewee 24, PTB)

Another woman stressed the importance of social support and how not having a support system makes it harder for people to overcome challenges.

## Discussion

As a qualitative study, this research provided a space for women to share their experiences of healthcare discrimination and how they recover from these experiences, as well as other stressful periods in their lives. Findings from this study highlight experiences of healthcare discrimination during pregnancy and childbirth among AA/BL postpartum women. Notably, most instances of healthcare discrimination during pregnancy and childbirth are subtle and manifest in inadequate quality care, such as dismissing health concerns, not being listened to, and poor communication from providers, which some attributed to race. These findings align with prior research that have identified patterns of healthcare discrimination and inadequate care during pregnancy [30, 31, 33, 56–63]. In addition to experiencing healthcare discrimination themselves, women in our study also reported negative assumptions about their partner and/or the father of the baby. These findings were consistent with the literature. Janevic et al. (2020) found that Black and Latina postpartum women reported differential treatment based on the father of the baby [58]. These findings from the literature and our study underscore the need for further research on experiences of healthcare discrimination not only among AA/BL women but also among their partners and/or fathers of the baby, as well as the potential impact this has on healthcare during pregnancy and pregnancy outcomes.

Moreover, it is evident from the interviews that experiences of healthcare discrimination during pregnancy and childbirth are a significant source of stress. The accounts of perceived discrimination underscore the importance of high-quality care, which for AA/BL women is often viewed as feeling respected, cared for, and listened to by healthcare providers. When these expectations are not met, it can lead to feelings of dismissal and fear, contributing to overall stress. Therefore, as healthcare systems work to improve access to care during pregnancy, the treatment received while accessing that care is equally important and can significantly impact future healthcare interactions and pregnancies [60]. Our findings further highlight structural racism in women’s healthcare [64, 65] and the underlying institutional and cultural practices that require intervention. Future research should explore these experiences of healthcare discrimination and their impact on stress and pregnancy outcomes among AA/BL women. Additionally, using implementation methods [66], researchers should explore clinical interventions that seek to address health discrimination and improve culturally responsive care during pregnancy and childbirth. Interventions that take a trauma-informed approach may help to address the ongoing inequities in healthcare that AA/BL women face [67].

As a response to experiences of healthcare discrimination, AA/BL women leveraged resilience in several ways. First, women sought Black healthcare providers to ensure positive healthcare experiences during pregnancy and to mitigate experiences of discrimination and poor-quality care. These findings were consistent with another qualitative study that found Black birthing people preferred racially concordant providers to reduce adverse outcomes and as a result of experiencing racial discrimination throughout their lives and in healthcare [38, 68]. Women in our study shared similar experiences of not being heard, stereotyping, and advocating for themselves as a strategy to avoid harm during pregnancy and childbirth [38, 68]. Teal et al. (2025) found that despite the preference for racial concordance with obstetricians and midwives, there was difficulty in finding racial concordant providers [38, 68]. Considering that these studies were conducted in very different parts of the U.S, these findings underscore the need for healthcare systems to improve the diversity of the obstetric healthcare workforce across the nation. Further, women demonstrated resilience through taking action. Women discussed reported filing complaints, which could be a source of information for health care institutions to consult in considering ways to combat healthcare discrimination. Our study adds to the literature by illustrating interpersonal factors that can be used to establish better provider-patient relationships and address the interpersonal discrimination among AA/BL women during care. It should be noted that while highly resilient, AA/BL women should not be required to cope with experiences of healthcare discrimination when seeking care during pregnancy. However, this study proposes valuable insights into the resilience of AA/BL women during pregnancy and thus should be used to design strength-based interventions among AA/BL women at risk for APOs.

The second way that AA/BL women demonstrated resilience was through strength and determination, and the will to keep going. Strength and determination among AA/BL women have been explored in the literature as the “strong Black woman” or “superwoman” phenomenon. Researchers have explored the superwoman phenomenon using the Superwoman Schema [SWS] framework, highlighting how AA/BL women under sociopolitical and sociohistorical contexts cope and survive oppression [69]. Patterns of SWS were seen in our study. AA/BL women displayed strength and determination, which was displayed through not giving up and having optimism. It was common for women to prioritize being strong and independent when faced with life stressors and pregnancy-related stress. In addition, AA/BL women prioritized the needs of their children and the need to be strong for them. Additionally, we found that AA/BL women use healthy coping mechanisms which included prayer, journaling, listening to music and self-care, to build resilience and overcome challenges and stress in their lives. Furthermore, as found in the literature [33, 51], we found that social support and spirituality are common resilience promoting factors among AA/BL women. Coping strategies and resilience promoting factors identified in this study can be used to explore and develop culturally tailored stress reduction and resilience interventions for AA/BL women in pregnancy. Moreover, these healthy coping mechanisms may or may not decrease adverse health outcomes. The SWS has been coined a “double-edged sword” for AA/BL women, as it has been found to be protective against adverse health outcomes but also may lead to stress and adverse health outcomes [48, 70, 71]. Thus, future research should explore the SWS and ways that AA/BL women cope and its influence on APOs among AA/BL women.

Our study is the first study to explore experiences of healthcare discrimination and resilience among AA/BL women with different pregnancy outcomes. While our study did not specifically explore differences in experiences across pregnancy outcomes, findings did not show any clear differences across groups. These findings may suggest that for AA/BL women, healthcare discrimination is widespread and affects AA/BL women regardless of clinical outcomes, further highlighting the pervasive impact of healthcare discrimination among AA/BL women and the need for immediate intervention. Future research should continue to explore differences in experiences and coping responses among AA/BL women with different pregnancy outcomes.

### Strengths and Limitations

Strengths of this study include using a purposive sample of AA/BL women from different socioeconomic statuses, ages, and pregnancy outcomes to gain a diverse perspective of experiences of perceived healthcare discrimination and resilience. Additionally, interviews were conducted by a Ph.D. candidate who identifies as AA/BL. Thus, given that women were also AA/BL women, this may have influenced responses. It is also important to highlight that the interviewer also had experiences of not being listened to during pregnancy and experienced a PTB, which can lead to bias in the development of research and thematic analysis. Reflexivity and debriefing with the senior qualitative researcher who did not have these experiences, as well as others on the research team, helped to mitigate these biases. This study is one of few of qualitative studies that has aimed to amplify the voices of AA/BL postpartum women, which are often unheard. Thus, women’s stories were used to highlight the injustices they experience and their strength to keep going. This study also includes a few limitations. Our sample size was only 30 AA/BL women, and they were all recruited from the same hospital and region, which may not fully capture the diverse experiences of women receiving care at other facilities and outside of Milwaukee, WI. Further, this study was conducted in one of the most highly segregated cities in the United States [72]. Considering this, AA/BL women in this study could have a heightened awareness of discrimination which may have impacted experiences.

## Conclusion

AA/BL women described experiences of healthcare discrimination through poor quality of care during pregnancy and childbirth. As a result of historical and present-day healthcare discrimination, women sought Black clinicians during pregnancy in hopes of mitigating these negative experiences. AA/BL women in this study demonstrated strength and resilience despite having experienced healthcare discrimination and described effective strategies for coping and building resilience. Future research should explore interventions to eliminate healthcare discrimination to advance health equity in maternal health.

## Data Availability

The data supporting these findings are not publicly available due to the confidentiality of participants. De-identified data may be available from the corresponding author upon reasonable request, and is subject to IRB approval.
